# Both optical and rheological properties contribute to viscosity judgements when comparing real liquids using vision and touch

**DOI:** 10.1098/rsos.241170

**Published:** 2025-02-26

**Authors:** Jeffrey Martin, Matjaž Jogan

**Affiliations:** ^1^ Johnson and Johnson Consumer Inc., 199 Grandview Road, Skillman, NJ, USA; ^2^ University of Pennsylvania, 3400 Spruce Street, Philadelphia, PA, USA

**Keywords:** psychophysics, perception, viscosity, Weber fraction, haptic, visual

## Abstract

Does the opacity of a liquid, or the way it reflects light, affect how viscous it appears? Would the perceived viscosity change if we touch the liquid? Viscosity of a material, or how it flows, produces a rich stimulus that reflects the material’s rheological properties, which can be independently inferred by touch or vision, or by combining modalities. In addition, the material’s optical properties convey other cues not dependent on rheology, such as colour or transparency. How these multisensory cues form stable percepts of viscosity that drive behaviour remains unknown. To shed light on this mapping, we conducted a series of psychophysical experiments in which observers compared the thickness of real liquids. First, we show that perceptual discrimination tends to scale in proportion to stimulus viscosity. Next, we show that optical properties such as transparency and opacity, although not related to viscosity, influence visual judgments of thickness. This bias, driven by appearance, is reduced when observers combine visual and haptic information. Combining information across visual and haptic senses improves discrimination only over a limited viscosity range.

## 1. Introduction

The physical properties of objects and stuff we interact with in our daily lives are easily perceived by our senses. We can often recognize a material, judge its colour and texture and assess the physical properties important for manipulation. For example, is it liquid or solid, wet or dry, hot or cold? One material property we frequently encounter is the viscosity of a flowing material, often referred to colloquially as ‘consistency’ or ‘thickness’ and defined physically as resistance to flow. Viscosity results from the interaction of the individual components within a material when it is forced to flow. For instance, the viscosity of water is relatively low compared to that of motor oil because water molecules are smaller and more mobile than larger hydrocarbon molecules in lubricating oil. Humans seem to reliably predict, based on observation, that an aqueous liquid will flow differently than motor oil. Inferring physical dynamics from sensory data is therefore one of the functions for which our sensory apparatus must be adapted.

Viscosity and other material properties are critical to the performance of important man-made materials such as coatings, paints and lubricants. In the formulation of other man-made materials, such as food and personal care products, these properties are usually considered, for example, by tuning viscosity and other physical properties to simultaneously create a stable formulation and a pleasant consumer experience. This requires not only a detailed knowledge of the dynamics of a material’s rheological properties, but also an understanding of how these properties affect the perception of the material during use. Much research has been done on the perception of solid-like properties such as compliance [1–8] and somewhat less on the perception of liquid-like materials [9–12], which we address in this work. The perception of materials in the viscoelastic range, where materials behave neither strictly as solids nor as liquids, has not yet been adequately explored, largely because of the increasing dimensionality of material properties and their dependence on the nature of deformation. The attempts in this area that are limited to linear deformations have limited applicability in real situations [13] or are focused on linking perception to sensory attributes [14,15]. While perception of viscoelastic materials is an interesting and important topic, in this work we avoid any confounding effects of viscoelasticity by carefully choosing materials which exhibit strictly viscous responses to deformations.

The most obvious cue to viscosity is the haptic resistance of the material. Physically, shear viscosity is defined as the ratio of stress to shear rate. This resistance creates a tactile sensation whenever we interact with a fluid material, either directly or indirectly when we use tools to stir, distribute or dispense it. The haptic sensory pathway senses this resistance using both cutaneous and kinesthetic information [9]. Early studies on the haptic perception of fluid properties found that attributes, such as ‘wet’, ‘textured’, ‘silky’ and ‘viscous’, all accessible through touch, contribute to the discrimination of fluids [14]. Tiest *et al*. [10] provide a comprehensive review of this work and compile Weber fractions that represent the change in stimulus intensity (here, viscosity) needed to perceive a just noticeable difference (JND), relative to the original intensity, for haptic sensing across a wide range of viscosities and methods (manual and oral manipulation). When liquids were stirred with a finger, the Weber fraction for viscosity discrimination depended on viscosity magnitude, ranging from about 5 at a viscosity of 200 cP to about 0.3 at a higher viscosity of 16 000 cP [10]. This result led Tiest *et al.* to conclude that haptic viscosity discrimination is not described by Weber’s law, which states that the ability to perceive differences is proportional to the stimulus magnitude. At lower viscosities Weber fractions increased, implying that absolute discrimination thresholds are approximately constant and Weber fractions are inversely proportional to reference viscosity [10].

Viscosity also affects the shape of the material and how it unfolds in time. Low viscosity fluids tend to flow easily, yield to gravity by dissolving and flattening their volume and produce relatively large numbers of droplets when sprayed. High viscosity fluids flow slowly and resist gravity by maintaining a thicker and more compact shape longer compared to thinner fluids. Therefore, for the same driving force, the velocity of local flow is generally higher for less viscous fluids than for thicker fluids. Visual cues presented to the observer describe a complex interaction of shape and motion which needs to be disentangled to judge viscosity across different contexts. This could be achieved through ‘intuitive physics’ [16,17], i.e. by simulating an internal model of the world that captures the rules of material motion to infer the physical parameters. Van Assen *et al*. [18] propose an alternative hypothesis where the visual system achieves stable judgements of viscosity using a combination of mid-level features that describe the material spatial distribution, irregularity, straightness and dynamics. Although some studies suggest that two-dimensional features, such as colour and texture, micro-texture, outline shape and reflectance, are sufficient for material property recognition [19], and viscosity can be judged surprisingly well when viewing static snapshots [20] or from two-dimensional optic flow [21], it appears that viscosity is more robustly perceived using mid-level features representing the dynamics of the three-dimensional surface.

Beyond shape and motion, visual cues also reflect the optical properties of liquids. These properties are independent of viscosity and would be discounted if the objective was to achieve a veridical judgement. However, as the visual input is noisy and uncertain, considering the optical properties could also enhance perception if prior experience with similarly looking liquids was taken into account [22]. However, recent evidence based on perception of artificially generated stimuli suggests that the effect of optical properties on the perceived viscosity is small. In a recent study [23], noise and factors other than viscosity accounted for less than 1% of total variance in a linear regression model, suggesting that, when reliable motion and shape cues are available, the visual system can estimate viscosity without biasing the estimates based on appearance. The artificial stimuli in [23] were made to mimic the appearance of opaque and transparent liquid materials such as caramel, metallic car paint, milk, water and wine; authors found that the effect of appearance on perceived viscosity was low compared to other cues even for stimuli as different in appearance as milk (opaque) and water (transparent).

In this work we attempt to characterize perception of viscosity from a multimodal perspective and using real materials, measuring perception constructed from visual information that emanates from materials of varying optical properties, haptic information and the combination of both. We limit ourselves to materials that only exhibit viscous nature and no shear-dependent or time-dependent behaviour, meaning they are Newtonian and non-thixotropic (§2). We attempt to answer three basic questions: (i) How does human sensitivity to viscosity differences vary across viscosity ranges with the use of vision and touch, separately? (ii) To what degree is perception of viscosity dependent on the optical properties of real fluids, in particular by their opacity? and (iii) How does perception change when observers combine visual and tactile information?

Our work is the first attempt to approximate real-world conditions by measuring the visual and haptic perception of a unique set of physical materials to quantify perception thresholds and bias for perception of viscosity in multiple modalities and with fluids of differing visual character. In a set of psychophysical tasks, we first confirm that viscosity discrimination does not always follow Weber’s law across the viscosity spectrum, but only with visual transparent stimuli. Next, we show that visual perception of viscosity might be significantly influenced by how opaque the liquid is, with observers, on average, not discounting the optical properties that are not related to viscosity. Finally, we discuss potential reasons for a more veridical perception of viscosity in a multimodal setting where participants experience viscosity simultaneously through haptic and visual means.

## 2. Materials and methods

**Stimulus preparation** Shear viscosity is defined as the ratio of shear stress to shear rate:


(2.1) $$\eta ≝\frac{\Sigma }{\overset{˙}{\gamma }}$$

where Σ is the shear stress and $\dot{\gamma }$ is the shear strain rate, or equivalently the shear rate. The steady-state viscosity response of flowing materials can be single-valued and constant with shear rate (Newtonian), or the viscosity can vary with the shear rate (shear thinning/shear thickening). The viscosity response of a material can also be time dependent and shear history dependent (thixotropy). To ensure a constant stimulus viscosity during manipulation, where the flow fields and history can become quite complex, we limited our stimulus set to non-thixotropic, Newtonian fluids. Fluids were mixed from silicone oil (polydimethylsiloxane) viscosity standards of 100, 500, 1000, 5000, 10 000 and 30 000 cP (Brookfield Engineering, Middleboro, MA, USA) to create 21 stimuli with viscosities ranging from 150 to 30 000 cP shown in table 1 along with some illustrative examples of common materials exhibiting similar viscosities. For example, sample five from table 1 was prepared by mixing 781 g of the 500 cP silicone oil viscosity standard with 209 g of the 1000 cP silicone oil viscosity standard, giving 990 g total and a resulting viscosity of 620 cP. Adjacent standards were always used to make the final fluids in order to keep the molecular aspects of the fluids (primarily molecular weight) as similar as possible; for example, samples 1–3 were generated by blending the 100 and 500 cP viscosity standards, samples 4–6 by blending the 500 and 1000 cP standards, 7–13 by blending the 1000 and 5000 cP standards and so on. Opaque fluids were created by adding a small amount (less than 1% by weight) of a silicone-coated zinc oxide to aliquots of each of the 21 fluids, giving them a white, fully opaque appearance with no perceivable texture. The viscosity of the fluids increased slightly with the addition of the zinc oxide particles while still retaining Newtonian behaviour, which is expected due to the increased hydrodynamic interaction of the particles. To ensure Newtonian behaviour, viscosity values were determined by performing a steady-state flow curve on a TA Instruments ARES G2 rheometer equipped with a 50 mm diameter, 1 degree cone and plate geometry. The steady-state flow curve was performed by stepping the shear rate from 0.1 to 1000 s^−1^, where a steady-state algorithm ensured that the measured viscosity value at each shear rate was constant before moving on.

**Table 1.** Measured viscosity values of the silicone oil fluids used in the studies.

	viscosity (cP)		
sample ID	transparent	opaque	approximate example
1	153	152	
2	179	202	
3	272	306	
**4**	**547**	**544**	orange juice concentrate
5	620	687	
6	756	827	
**7**	**1040**	**1110**	motor oil SAE 60
8	1130	1230	
9	1290	1420	
**10**	**1560**	**1710**	laundry detergent
11	1930	2140	
12	2580	2980	
**13**	**3520**	**4050**	molasses
14	5520	5870	
15	7060	7420	
16	9480	10 630	
17	12 950	14 140	
**18**	**15 530**	**16 850**	caramel
19	18 930	20 440	
20	22 780	25 650	
21	31 160	33 810	

Video clips of fluids were generated by recording videos using a Nikon D750 SLR camera with a Nikkor AF-S 105 mm F/1.4 lens, on a tripod, set to video mode and capturing in greyscale at 60 fps. The camera was aimed at a setup with controlled light conditions with two video lights illuminating the scene. The setup consisted of a mechanical actuator, a stand and gripper for a dispensing bottle and a rigid, slanted plastic sheet with a textured surface. After filling each dispensing bottle with the corresponding liquid, the actuator pressed the nozzle pump with a steady speed of 1 cm s^−1^ for 1.4 cm, which corresponded to near the maximum travel distance of the pump nozzle. After reaching the low point, the direction of movement of the actuator reversed at the same speed. The nozzle was firmly attached to the actuator and always travelled to the initial position before the cycle was repeated. Each video was generated by recording three consecutive down/up pump cycles for each liquid, and then subsequently cropped and synced in Adobe Premiere. The duration of the videos was 11 s. The full set of videos is available as supplementary material.

We utilized a typical cosmetic packaging bottle and pump combination; therefore, it is prudent to check how experience with cosmetic products falls within the viscosity range of stimuli that we created and whether a different distribution of viscosities of opaque and transparent stimuli might introduce a strong association between viscosity of everyday products and appearance. To accomplish this, we measured 44 currently marketed personal care products (31 opaque and 13 transparent) with identical or very similar dispensing pumps and nozzle shapes. The average shear rate in the nozzle was determined to be approximately 200 s^−1^ from the average volumetric amount of product dispensed per pump, the pump actuation speed and the nozzle diameter (5 mm by 2.5 mm). The distributions of viscosities at 200 s^−1^ for these 44 representative products will be discussed later. We found no products in our search which exhibited a viscosity above about 2300 cP at a shear rate of 200 s^−1^.

### 2.1. Experimental paradigm

For all studies, a two-alternative forced-choice (2AFC) procedure was utilized with the method of constant stimuli. In a 2AFC task, participants were presented with a test and a reference stimulus simultaneously (in the visual tasks) or sequentially (in the haptic tasks). The task was to choose the stimulus that is thicker, and then move on to the next stimulus pair. Participants were presented with each test-reference pair in each group before moving onto the next group. Left-right placement of the test and reference stimuli, the order of test-reference pairs within each group, and the order in which the groups were completed was randomized across all participants. Each stimulus pair was presented to each participant once.

### 2.2. Experimental setup

Three separate viscosity perception experiments were performed as described in the following sections: a visual perception study, a haptic perception study and a mixed modality visuo-haptic study (table 2). In the visual study, we determined Weber fractions for visual perception of viscosity for transparent and opaque liquids (tasks 1a and 1b, respectively), as well as the point of subjective equality (PSE, the point at which the viscosity of the transparent test stimulus is perceived to be equal to the viscosity of the opaque reference stimulus) between transparent and opaque liquids (task 1c). Weber fractions for haptic perception were similarly determined in the haptic study (task 2) and subsequently for a mixed modality visuo-haptic study utilizing the transparent liquids (task 3a). Finally, the PSE was determined for the mixed modality task (task 3b).

**Table 2.** List of tasks across studies.

study	task	modality	trials	reference	test	*N*	measured parameter
**visual**	**1a**	visual	32	T	T	57	Weber fraction
	**1b**	visual	32	O	O	57	Weber fraction
	**1c**	visual	35	O	T	110	bias (PSE)
**haptic**	**2**	haptic	32	T	T	64	Weber fraction
**visuo-haptic**	**3a**	mixed	32	T	T	50	Weber fraction
	**3b**	mixed	32	O	T	50	bias (PSE)

T, transparent; O, opaque.

#### 2.2.1. Tasks 1a, 1b and 1c (visual)

Participants were recruited from a cohort for consumer testing of skincare products. Exclusion criteria were dictated by internal company standards and are as follows: pregnant or planning to become pregnant in the next 30 days; is breastfeeding; have participated in any skincare consumer or clinical study in the past 30 days; works in market research, advertising, pharmaceutical industry or skincare-related field; history of taking medications related to diabetes, skin cancer or skin conditions such as eczema; self-described as having excessively dry skin; currently have injuries on the fingers or hands; has known allergies or sensitivity to skincare products; has known allergies or sensitivity to silicones, silicone-containing materials or polydimethylsiloxane polymers. After arriving at the testing site, participants (*n* = 114) signed the consent form while a study administrator served as witness and were then briefed on the task by a study administrator. They were randomly assigned to either observe transparent (task 1a, *n* = 57) or opaque (task 1b, *n* = 57) stimuli and seated behind a desk in a dedicated cubicle with subdued lighting to minimize screen reflection. Stimuli were presented on a 17″ LCD screen (Samsung), connected to an Apple Mac mini computer, using the stimulus presentation package PsychoPy [24]. The screen resolution was 1366 × 768 at 60 Hz. The screen was positioned 15 inches from the edge of the desk, at eye level, and participants were not constrained in looking at the screen. Pairs of stimuli were displayed (duration 11 s) and the participant had to choose the one that appeared thicker by pressing the left or right key on the keyboard. Each task started with a training phase of four pairs with large differences in viscosity and feedback on correct or incorrect choices. Participants who did not complete the four trials without error were excluded. In the testing phase, there were five groups of stimuli (A–E, see table 3); participants viewed seven trials in groups A and E, and six trials in groups B–D, as these groups could use overlap pairs (e.g. the test/reference pairing of 1710/1110 cP appears both in groups B and C), and each trial was performed only once. There was no feedback given in testing. All but four (*n* = 110) participants also completed task 1c where the reference and test stimuli had a different appearance. The four participants were excluded as they did not pass the training phase. Participants viewed seven trials in all groups A–E, as due to different appearance there was no overlap in stimuli in task 1c. For each participant, the order of the two tasks (1a or 1b, and 1c), the order of the five stimuli groups and the order of the stimuli pairs within each group were all randomized.

**Table 3.** Measured viscosity values in cP and groupings for transparent and opaque fluids.

	visual opaque					visual transparent and haptic				
	viscosity (cP)					viscosity (cP)				
	**A**	**B**	**C**	**D**	**E**	**A**	**B**	**C**	**D**	**E**
**test 1**	152	544	1110	1710	7420	153	547	1040	1560	7060
**test 2**	202	687	1230	2140	10 630	179	620	1130	1930	9480
**test 3**	306	827	1420	2980	14 140	272	756	1290	2580	12 950
**reference**	**544**	**1110**	**1710**	**4050**	**16 850**	**547**	**1040**	**1560**	**3520**	**15 530**
**test 4**	687	1230	2140	5870	20 440	620	1130	1930	5520	18 930
**test 5**	827	1420	2980	7420	25 650	756	1290	2580	7060	22 780
**test 6**	1110	1710	4050	10 630	33 810	1040	1560	3520	9480	31 160

#### 2.2.2. Task 2 (haptic)

Exclusion criteria and pre-study activities were identical to the visual study. Participants were then seated behind a desk on which was placed a wooden box divided into two compartments with one hole for each compartment on the side facing the participants, the holes being covered by dark fabric that allowed the participants to place their hands in the box without viewing the contents. For each comparison, the study administrators placed jars (7 cm tall by 5 cm diameter) containing the fluids into each compartment (one test and one reference).

Participants (*n* = 64) were instructed to place a latex finger glove (9 mil, 0.23 mm thickness) on the index finger of their dominant hand and stir the liquid in one jar with the covered finger. They then removed the finger glove, replaced it with a new finger glove, then stirred the other fluid. For each pair, the participant selected which sample was ‘thicker’ with a keypress. Left/right placement of samples, order of sample groups and pairs sequence within each group were randomized, and each individual trial was performed only once.

#### 2.2.3. Tasks 3a and 3b (visuo-haptic)

Exclusion criteria and pre-study activities were identical to the visual study. A total of *n* = 50 participants were recruited and assigned to complete tasks 3a and 3b, in random order. They were seated behind a desk with a haptic setup identical to the haptic study. In addition, visual stimuli were displayed on a screen in front of the participant as in the visual study. Pairs of stimuli were displayed and looped until the participant picked the one that appeared thicker by also being able to probe the stimuli with a finger as in the haptic study.

### 2.3. Ethics statement

Study protocols were reviewed and approved by IntegReview IRB, IRB00008463. Prior to participation, all participants signed a consent form while a study administrator served as witness.

### 2.4. Data analysis

For analysis, we aggregate the responses for each stimulus pair over all participants, therefore approximating a population-level psychometric function of the ‘average’ participant.

To quantify the discrimination threshold, the frequency of the ‘thicker’ response for each test stimulus (over all viscosities η of test stimuli in the group) is fit with a psychometric function:


(2.2) $$f\left(\eta \right)=0.5+0.5\;erf\left(\frac{\log\left(\frac{\eta }{p}\right)}{\sqrt{2}\log\left(w+1\right)}\right),$$

where η and *p* are the test and reference viscosities, respectively, erf(*x*) is the error function and *w* is a fitting parameter that determines the slope.

For each task and for each stimulus group centred around a reference stimulus, the function in equation (2.2) is fitted using a maximum likelihood procedure. Weber fractions and PSE for each reference stimulus can be extracted from the parameters, *p* as PSE and *w* as the Weber fraction. For tasks in which we measured Weber fractions, *w* was a fitting parameter. For tasks where we also measured PSE, both *p* and *w* were a fitting parameter. To prevent failures in fitting, parameter values were constrained to intervals *w*∈ [0.1, 100], *p*∈ [10, 20 500]. Initial values for fitting were (*w, p*) = (0.5, *p*_true_). For this function and the fitted *w*, the threshold for reliable discriminability is 84% [25]. Confidence intervals (CI) were obtained by bootstrapping [26]; for each test-reference pair, participant responses were sampled with replacement *N* times, where *N* equalled the number of task participants, thus creating a random sample from the response distribution. This random sample was then fit with the psychometric function. Resampling was repeated for 5000 iterations, each fit providing a pair of bootstrapped parameters *p* and *w*. Percentiles from the sorted bootstrapped values of *p* and *w* were used to construct the bootstrap CI and the mean. When assessing whether two independent parameters, such as bias or Weber fractions, are significantly different, we calculated the CI for the mean difference based on the bootstrapped parameters, reporting the mean difference Δ and the difference CI. When assessing the significance of bias measurements (PSE in tasks with different appearance of reference and test) we compare the bootstrapped interval of distances of PSE values to the true reference. We also report *p*-values derived from the distribution of differences between the two bootstrapped parameters distributions and correct for number of comparisons using Bonferroni correction. To confirm that bias is not an artefact of aggregating data across subjects, we calculate for each subject the percentage of ‘thicker’ responses for a particular stimulus set and compare the distributions using ANOVA and non-parametric tests.

We also compared whether distributions of viscosity of opaque and transparent cosmetic products on the market are similar or different. The difference in the two sets of measured viscosities was tested using the means difference CI, a two-sample Kolmogorov–Smirnov goodness-of-fit hypothesis test and the Wilcoxon rank sum test for equal medians.

## 3. Results

In the following sections, we report data on the perception of viscosity using vision, touch and a combination of vision and touch. We start with viscosity discrimination (visual tasks 1a, 1b and haptic task 2), continue with the result on bias due to appearance (task 1c) and end with discrimination and bias in a multimodal setting (tasks 3a and 3b). Raw data from all experiments is available at http://doi.org/10.5281/zenodo.10637378.

### 3.1. Perception of differences in viscosity with vision and touch

**Figure 1.**
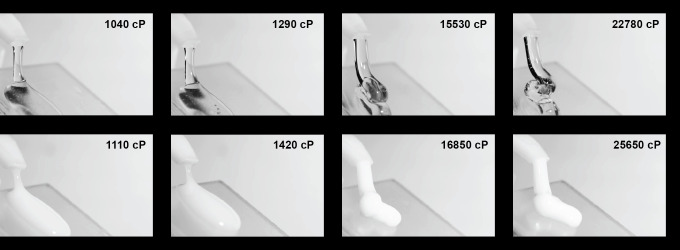
Still frames taken from videos showing four representative comparisons from the visual tasks: low/high viscosity for transparent and opaque liquids. Frames are taken at the point of maximum pump travel for the first of three successive pumps. Viscosity values are added for illustration and were not shown to participants during the discrimination task.

**Figure 2.**
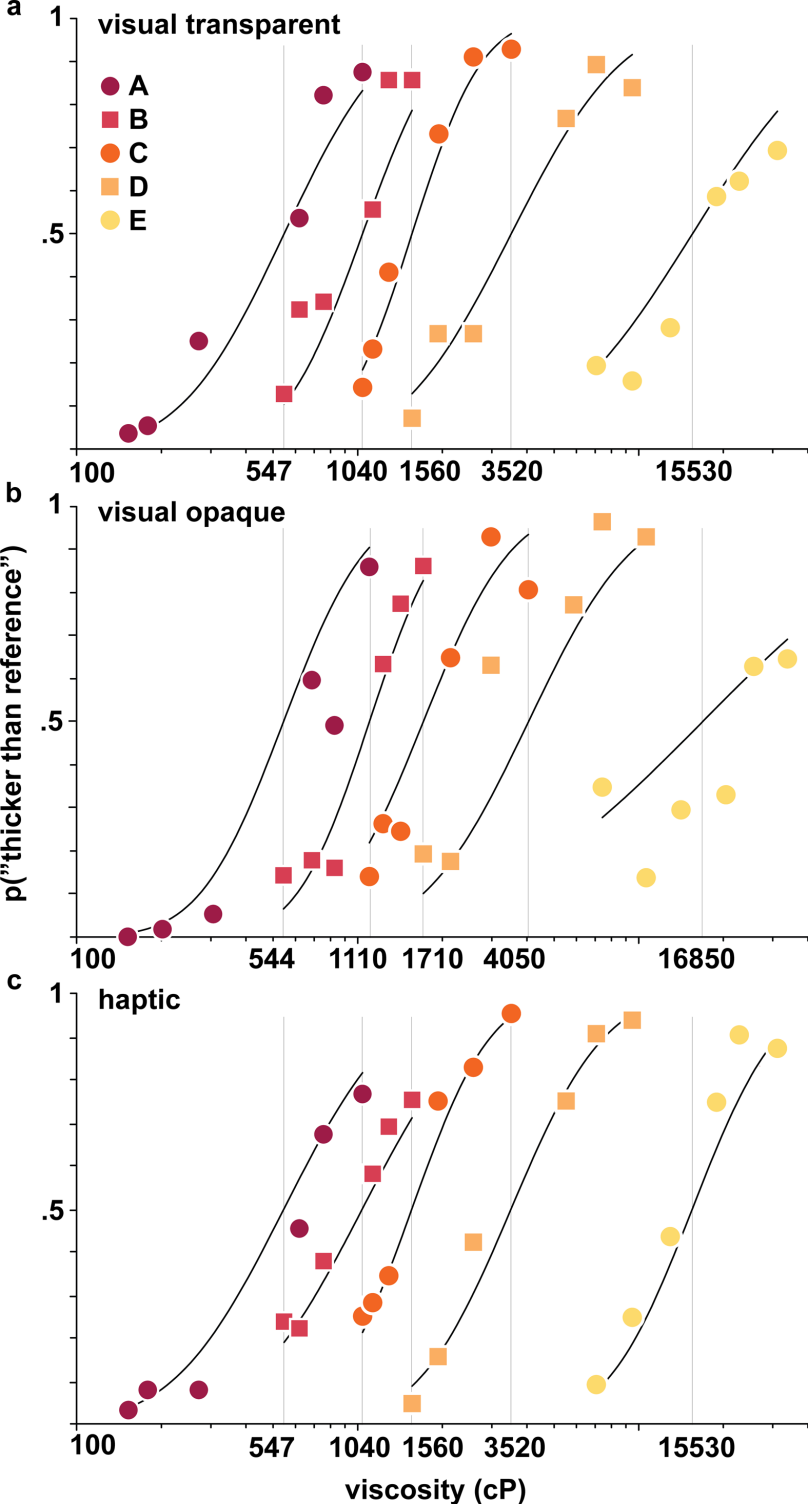
Psychometric curves plotted as the frequency of ‘thicker’ response versus viscosity for groups A–E from table 3 for (*a*) task 1a, visual transparent, (*b*) task 1b, visual opaque and (*c*) task 2, haptic modalities, on a logarithmic viscosity axis. Solid lines are fits of equation (2.2) with *p* set to true reference viscosity. Vertical lines denote the viscosity of the reference stimuli.

Both visual stimuli (examples shown in figure 1) and haptic stimuli were separated into five groups (A–E) (table 3), and perception was measured as a function of the central stimulus in each group. The aggregated responses from the visual discrimination and haptic studies are shown in figure 2 with corresponding fits of a psychometric function from which we extracted the population level Weber fractions. For the visual modality with transparent and opaque fluids, discrimination was difficult for the participants at high reference viscosity, as evidenced by shallow slope of the psychometric function for group E (transparent) and the variability in the responses for group E (opaque), resulting in a poor fit of the psychometric function. Fitted Weber fractions for all groups within both modalities are listed in table 4 and define the JND for each reference stimulus as $\frac{p}{\left(1+w\right)}$ and $p\left(1+w\right)$, where *p* is the reference viscosity and the JND corresponds to a discriminability of 84% (16%) [25]. CI for Weber fractions were from bootstrap as described in §2. We only consider results for which the 95% CI for the Weber fraction was smaller than 2 units. This criterion was set based on the expected values for Weber fractions and post hoc confirmed based on the distribution of CI widths where a width larger than 2 was a clear outlier. Thus, a Weber fraction is not listed for the visual modality with opaque fluids in group E (16 850 cP reference viscosity).

**Table 4.** Calculated Weber fractions with corresponding 95% CI.

modality	reference viscosity (cP)	Weber fraction	95% CI
**visual** dispensing transparent	547	**1.00**	0.79–1.29
	1040	**0.78**	0.60–1.07
	1560	**0.57**	0.45–0.73
	3520	**1.05**	0.82–1.38
	15 530	**1.43**	1.01–2.24
**visual** dispensing opaque	544	**0.87**	0.54–0.96
	1100	**0.61**	0.46–0.77
	1710	**0.77**	0.59–1.02
	4050	**1.06**	0.75–1.27
	16 850	**N/A**	N/A
**haptic**	547	**1.05**	0.84–1.35
	1040	**1.08**	0.79–1.61
	1560	**0.66**	0.53–0.84
	3520	**0.82**	0.68–1.02
	15 530	**0.74**	0.59–0.93
**visuo-haptic**	547	**1.29**	0.96–1.77
	1040	**0.86**	0.63–1.24
	1560	**0.63**	0.50–0.79
	3520	**0.54**	0.43–0.66
	15 530	**0.62**	0.51–0.86

**Figure 3.**
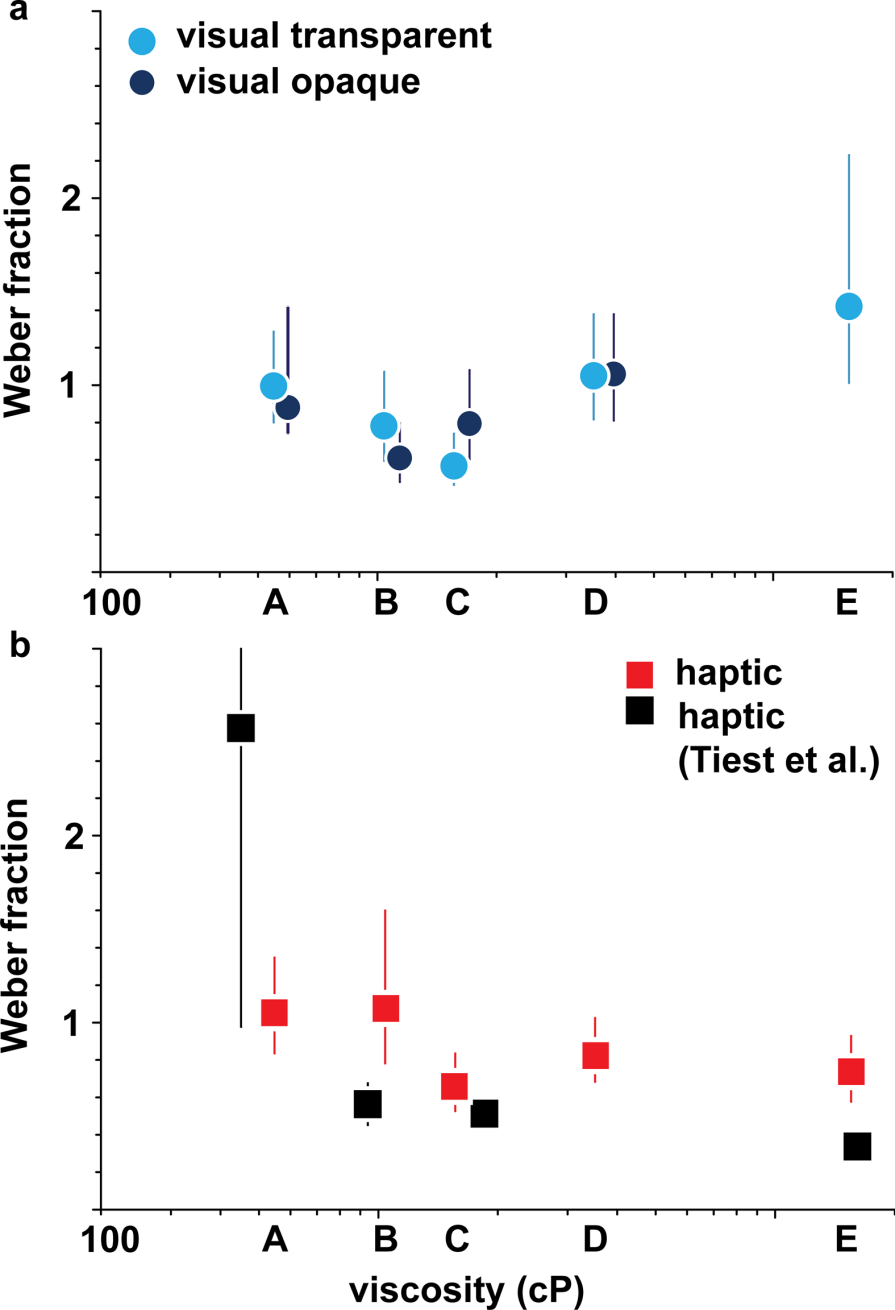
Weber fractions at reference viscosities for (*a*) tasks 1a and 1b, visual and (*b*) task 2, haptic modalities. For comparison we also plot the results from Tiest *et al*. [10].

Weber fractions are plotted against reference viscosity in figure 3 (refer to table 3 for reference viscosities per group). After Bonferroni correction for five comparisons (α = 0.01), none of the differences in Weber fractions between opaque and transparent stimuli were significant (544 and 547 cP: Δ−0.05, 99% CI [−0.53, 1.01], *p* = 0.71; 1100 and 1040 cP: Δ−0.18, 99% CI [−0.62, 0.18], *p* = 0.20; 1710 and 1560 cP: Δ0.23, 99% CI [−0.11, 0.68], *p* = 0.09; 4050 and 3520 cP: Δ−0.01, 99% CI [−0.53, 0.52], *p* = 0.99). The observers were therefore equally able to discriminate viscosities in transparent and opaque conditions, even if the visual information available was vastly different. However, when comparing the Weber fraction for the same stimulus type across different viscosities, we did observe a few differences. After Bonferroni correction for pairwise comparisons (*n* = 10, α = 0.005), participants better discriminated between transparent visual stimuli at mid-range viscosities, with thicker stimulus 3520 cP having a larger Weber fraction than the less viscous stimulus 1560 cP (Δ0.49, 99.5% CI [0.10, 1.01], *p* = 0.0004), and the most viscous transparent reference stimulus 15 530 cP also having larger Weber fractions than less viscous stimulus 1560 cP (Δ0.91, 99.5% CI [0.27, 2.50], *p* < 10^–8^), while the thinner stimulus 547 cP had a larger Weber fraction than stimulus 1560 cP (0.44, 99.5% CI [0.05, 0.93], *p* = 0.0016). For opaque stimuli and for the haptic modality, however, there were no significant differences in discrimination after Bonferroni correction except between opaque stimuli 4050 and 1100 cP (Δ0.45, 99.5% CI [0.01, 0.99], *p* = 0.0036). Our results for the haptic modality trend similarly to those of Tiest *et al*., but with slightly higher thresholds at viscosities 1040 cP and above (all 99% CI intervals are non-overlapping). The more precise measurement of the Weber fraction at 547 cP compared to Tiest *et al*. might be due to a larger sample size in our tasks, or to other task specifics. The overall quantitative differences are likely due to a different experimental setup and a larger number of participants in this work with a better-distributed sample of the general population, although limited to females (Tiest *et al*.: 5 males, 3 females, age: 20–30 years; our cohort: 64 females, age 21–55, all naive consumers). Aggregating the data across subjects might have also contributed to a flattening of the fitted psychometric curves [27], resulting in higher measured Weber fractions. However, this flattening would only contribute to a relative offset of Weber fractions that is similar across conditions, as experiments where we measure Weber fractions are symmetric, constraining the PSE to coincide with the reference value (no bias). Our results provide some support that Weber fractions might not always be constant across the viscosity spectrum as predicted by Weber’s law, and we found this violation holds for transparent stimuli. Weber fractions for opaque stimuli and haptic stimuli however showed barely one statistically significant difference across the references.

### 3.2. Point of subjective equality for comparison of opaque and transparent stimuli

**Figure 4.**
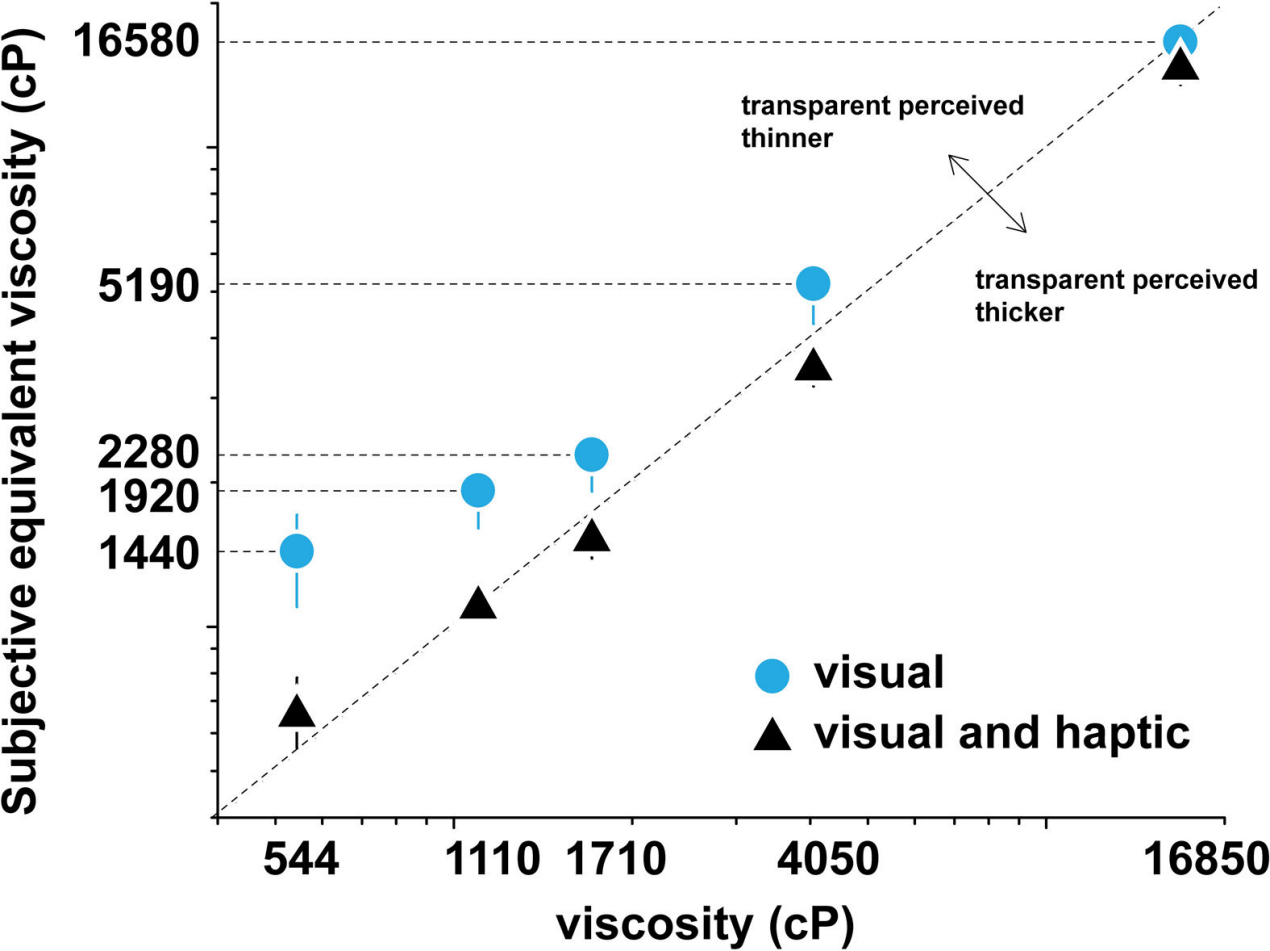
Subjective equivalent viscosity of transparent fluids versus opaque reference fluids, task 1c. For the visual modality, there exists a significant bias where transparent fluids are visually perceived to be much less viscous compared to opaque fluids of equal viscosity. Error bars indicate 95% bootstrap intervals.

In task 1c, 110 participants (four did not pass the training phase and were excluded) were shown dispensing videos of opaque (reference) and transparent (test) liquids side-by-side. As in tasks 1a and 1b, participants were instructed to choose the stimulus that appears thicker; the instructions did not mention the difference in opacity. By fitting the choice probabilities to the psychometric equation (equation 2.2), keeping *p* now as a free parameter, we could determine the PSE, i.e. the point where the participants judged the opaque and transparent liquids to have equal viscosity, thus revealing any potential biases due to liquid appearance. Figure 4 plots the PSE of the test (transparent) fluids against the opaque reference viscosity, measured as the difference of PSE to the reference viscosity. If optical characteristics of the stimulus did not affect the perception of viscosity, the PSE of the psychometric function for a particular group should equal the opaque reference viscosity and lie on the diagonal line. Any significant deviation from this diagonal line indicates a perceptual bias that might be driven by optical properties. The magnitude of this deviation is the magnitude of the bias.

We found a measurable perceptual bias between the transparent and opaque fluid viscosities. Figure 4 shows a very slight bias at high viscosity towards perceiving transparent liquids as more viscous than they are however the measurement is not significant after correction (Bonferroni correction for *n* = 5 comparisons, α = 0.01; 16 850 cP: Δ1667.17, 99% CI [−252.65, 3338.03], *p* = 0.013). More pronounced is the bias at lower viscosity. In the low viscosity range at or below 4050 cP, perception of transparent stimuli was decidedly biased towards lower viscosity values compared to opaque reference liquids: at 4050 cP: Δ669.00, 99% CI [122.10, 1310.61], *p* = 0.0004; at 1710 cP: Δ324.61, 99% CI [181.64, 491.20], *p* < 10^–8^; at 1110 cP: Δ643.78, 99% CI [427.45, 1033.50], *p* < 10^–8^; at 544 cP: Δ818.86, 99% CI [520.73, 1397.41], *p* < 10^–8^). Fits of the psychometric curve indicate that, for example, a participant would perceive an opaque liquid of viscosity 544 cP to be similar in viscosity to a transparent liquid of 1440 cP (nearly a factor of three difference). To illustrate this bias in terms of common materials, it is likely that a consumer would perceive the viscosity of orange juice concentrate (opaque liquid, roughly 600 cP) equal to liquid laundry detergent (transparent liquid, roughly 1500–3000 cP).

To verify that this bias is not caused by artefacts in this particular stimulus set, we tested perception on videos that were cropped to reveal only the upper portion of the scene, depicting the liquid dripping from the dispenser while hiding the information about the landing and flow over the plate. The results (*n* = 19, see electronic supplementary material, figure S1) confirmed that transparent liquids tend to be perceived as thinner. After excluding the psychometric fits using the same criteria as before (i.e. the width of the CI for the Weber fraction is larger than 2 units) we could only analyze bias at the three highest reference viscosities. Biases that were significant after Bonferroni correction (*α* = 0.01) were for references 1710 cP: Δ675.08, 99% CI [117.53, 2031.99], *p* = 0.0012, and 4050 cP: Δ1316.90, 99% CI [201.26, 2751.41], *p* = 0.0036, while bias at 16 850 cP was not significantly larger than 0, with Δ2863.40, 99% CI [−536.09, 3250.00], *p* = 0.0136. This result is similar to the bias obtained with original, uncropped videos.

Although aggregation over participants would not introduce bias if they had no bias, bias for the ‘average’ participant might have a magnitude and direction caused by a skewed underlying distribution of bias in the population. We thus verified that data had no obvious outliers (participants always choosing the reference or the test stimulus, participants always choosing left or right) that could heavily skew the per-participant bias distribution.

To further confirm that the observed bias is not an artefact of aggregating the data across participants, we compared the distributions of the percentage of responses for each subject and sample set where the test stimulus was perceived as thicker than the reference. We conducted an analysis of the differences between tasks, focusing on the comparison of mean percentages of responses in task 1c versus tasks 1a and 1b, and further comparing the results of task 1c with an expected 50% baseline in tasks 1a and 1b. These analyses were performed across five reference stimuli (A–E) and by separating the task 1c participants into two cohorts, depending on whether they observed transparent (task 1a) or opaque stimuli (task 1b). To assess the differences across tasks and cohorts, we employed both ANOVA and non-parametric tests. A Bonferroni correction was applied where necessary to account for multiple comparisons.

A three-way ANOVA was performed using task (two levels), cohort (two levels) and reference (five levels) as factors. The overall ANOVA revealed a significant main effect of task (*F*(1, 1090) = 421.997, *p* < 10⁻⁷⁹), suggesting a strong effect of stimulus appearance, and reference × task (*F*(4, 1090) = 19.392, *p* < 10⁻^16^), suggesting that the effect of stimulus appearance is not constant but depends on the viscosity range. The task × cohort interaction was not significant (*p* = 0.15).

For the cohort enrolled in task 1a (transparent), the Mann–Whitney *U* test revealed significant differences between task 1a and task 1c for most references after Bonferroni correction (*α* = 0.01). For Reference A, the difference in means was 21.64 (*U* = 2830.0, *p* < 10^–12^); for Reference B, the difference was 31.58 (*U* = 2946.0, *p* < 10^–14^); for Reference C, the difference was 17.93 (*U* = 2434.0, *p* < 10^–5^); for Reference D, the difference was 23.61 (*U* = 2755.0, *p* < 10⁻¹¹). For Reference E, the difference was not significant after correction (*U* = 1648.0, *p* = 0.76). However, Reference E was also the result we excluded from analysis due to a poor fit. The mean percentages in task 1c against an expected 50% baseline from task 1a were compared using the Wilcoxon signed-rank test. For this cohort, all references showed significant deviations from 50%. For Reference A, the difference was 38.01 (*W* = 1.0, *p* < 10⁻¹¹); for Reference B, the difference was 35.97 (*W* = 3.0, *p* < 10⁻¹¹); for Reference C, the difference was 17.35 (*W* = 36.0, *p* < 10⁻¹⁰); for Reference D, the difference was 12.50 (*W* = 136.0, *p* < 10⁻⁸); and for Reference E, the difference was 10.46 (*W* = 253.0, *p* < 10⁻⁶).

For the cohort associated with task 1b (opaque), significant differences between tasks were also observed. For Reference A, the difference in means was 25.88 (*U* = 2673.0, *p* < 10⁻¹³); for Reference B, the difference was 32.29 (*U* = 2764.0, *p* < 10⁻¹⁵); for Reference C, the difference was 20.91 (*U* = 2343.0, *p* < 10⁻⁷); for Reference D, the difference was 21.06 (*U* = 2372.0, *p* < 10⁻⁸); and for Reference E, the difference was 9.68, and this remained significant after Bonferroni correction (*U* = 1950.0, *p* = 0.0044, where α < 0.01). Significant deviations from the 50% baseline were also observed for all references. For Reference A, the difference was 33.07 (*W* = 3.0, *p* < 10⁻¹⁰); for Reference B, the difference was 31.75 (*W* = 6.0, *p* < 10⁻¹⁰); for Reference C, the difference was 15.34 (*W* = 120.0, *p* < 10⁻⁸); for Reference D, the difference was 19.84 (*W* = 55.0, *p* < 10⁻⁹); and for Reference E, the difference was 17.72 (*W* = 66.0, *p* < 10⁻⁹).

These results confirm that bias is not an artefact of data aggregation, as both the ANOVA and Mann–Whitney *U* tests demonstrate consistent differences in the percentage of responses between tasks, supporting the robustness of our findings across both cohorts.

**Figure 5.**
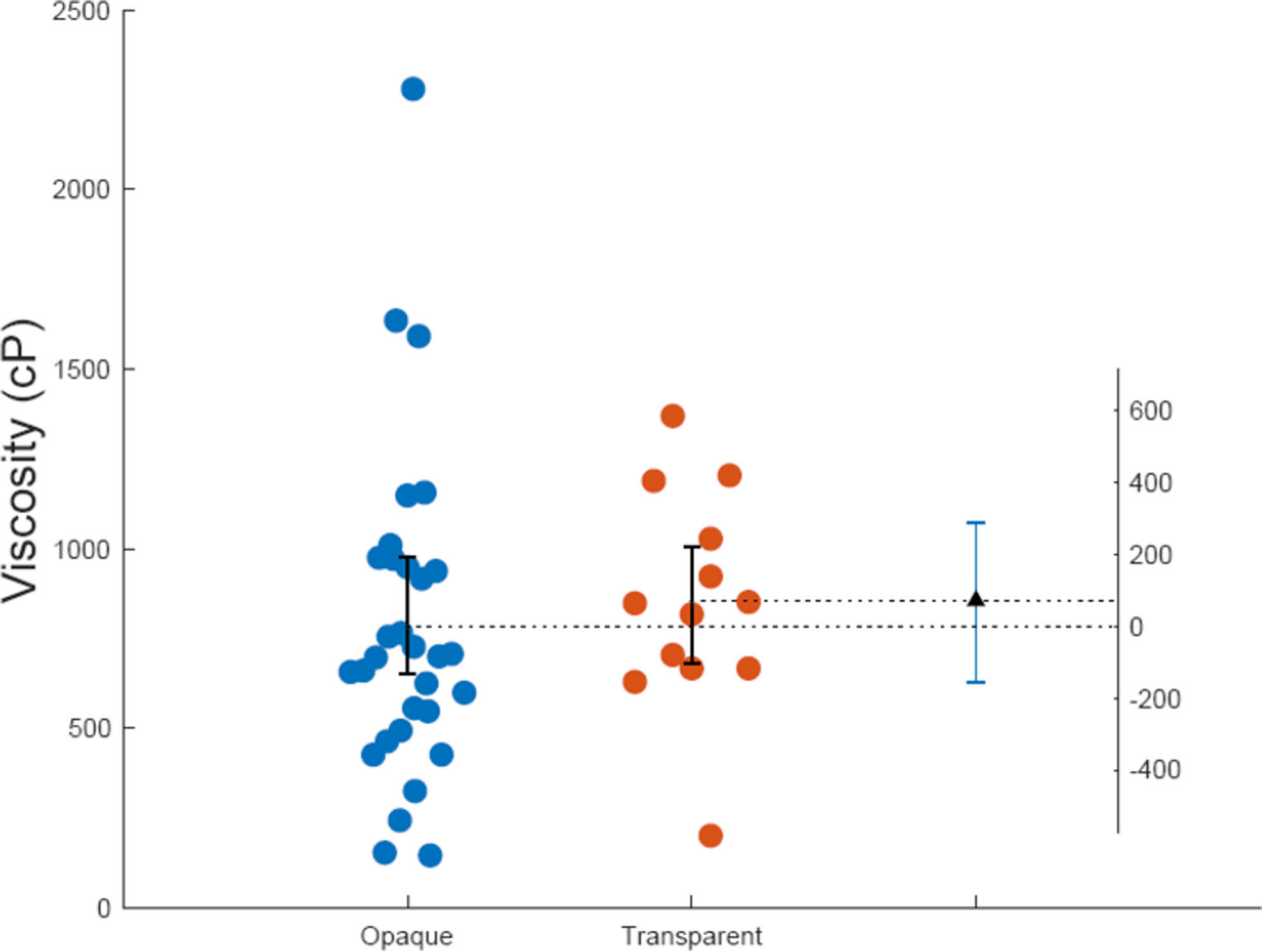
Viscosities at 200 s^−1^ for 31 opaque and 13 transparent personal care products having identical or similar dispensing nozzles to the ones used in this work.

Another source of information that might lead to the bias is the association of the pump or the dispensing action with a particular optical appearance based on prior use of cosmetic products (all participants were users of cosmetic products). We thus measured the viscosities of 44 personal care products widely available on the market: 31 opaque (mean = 782.75 cP, s.d. = 651.6) and 13 transparent (mean = 854.31 cP, s.d. = 682.92). Despite the observation that opaque products exhibit a broader range of viscosities (figure 5), statistical analyses comparing the distributions of the opaque and transparent groups did not demonstrate a significant difference. There was no difference between bootstrap means (Δ71.564, 95% CI [−155.14, 288.62], right *y*-axis in figure 5), and the two-sample Kolmogorov–Smirnov test for goodness-of-fit resulted in a *p*-value of 0.28 (*N*₁ = 31, *N*₂ = 13), suggesting no significant discrepancy in the distributions. Additionally, the Wilcoxon rank sum test for the equality of medians between the two sample sets yielded a *p*-value of 0.23 (*N*₁ = 31, *N*₂ = 13).

### 3.3. Combining visual and haptic perception

**Figure 6.**
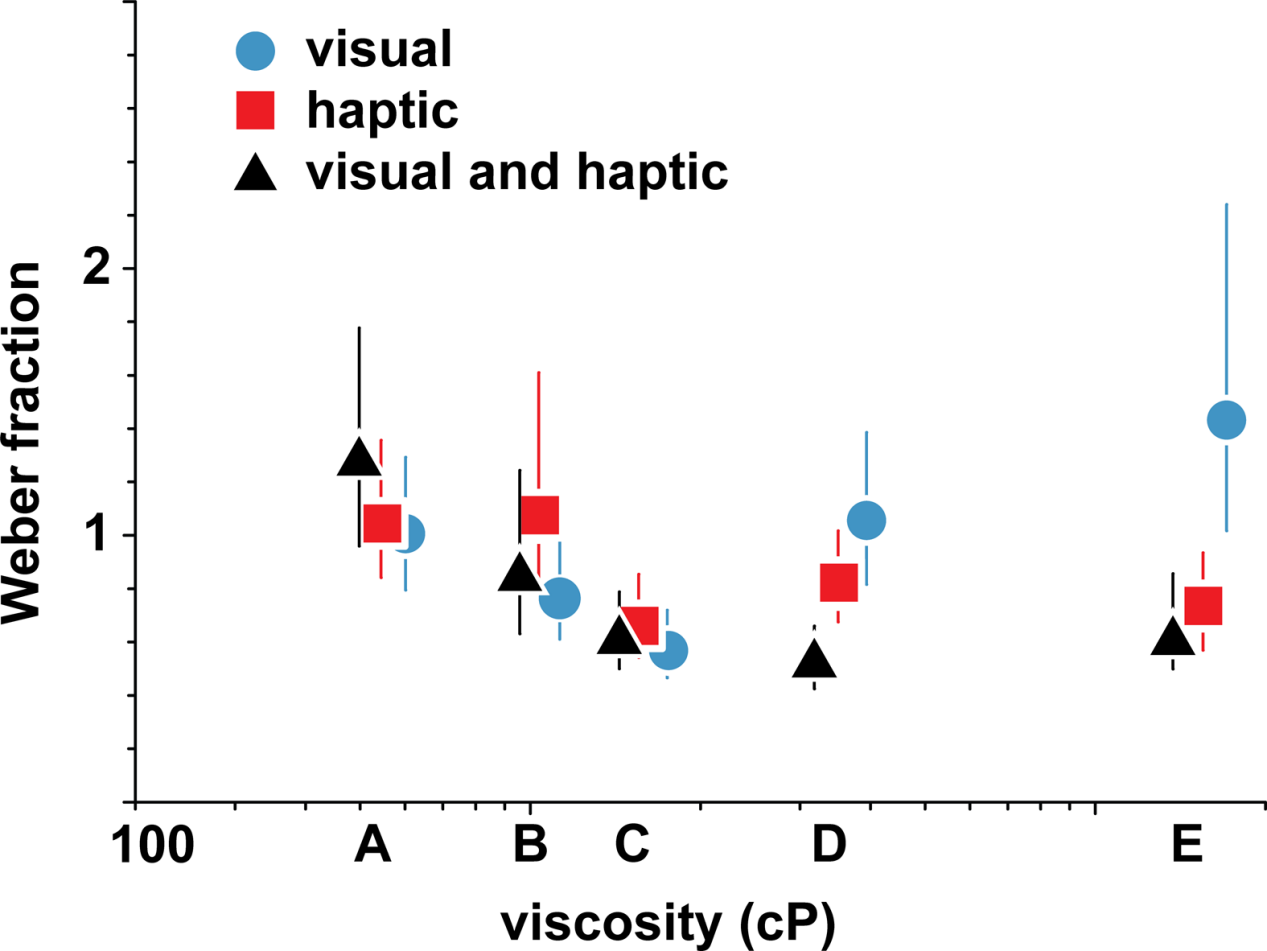
Weber fractions versus reference viscosity. Comparison of visual-transparent and multimodal stimulus Weber fractions. Error bars indicate 95% bootstrap intervals.

In the visuo-haptic study with tasks 3a and 3b, 50 female participants observed stimuli pairs in a multimodal setting. The videos were displayed on the screen as in the visual study, but now participants were also asked to simultaneously stir the same liquids that were occluded from view, as in the haptic study. In task 3a (combined visual and haptic for transparent fluids), both the reference and test *visual* stimuli were transparent. In task 3b (determining PSE between transparent and opaque liquids), the test visual stimuli were transparent while the reference visual stimuli were opaque. Resulting Weber fractions from the visuo-haptic study are shown in figure 6, along with Weber fractions from tasks 1a and 2 (the visual-transparent and haptic studies) and listed in table 4.

Surprisingly, the discrimination ability did not improve across all viscosity ranges when compared to the lowest values of Weber fractions for visual-transparent (task 1a) or haptic (task 2) discrimination. Compared to the lowest of the two Weber fractions at a particular reference viscosity, the multimodal Weber fraction was significantly lower only against the haptic-only perception of viscosity 3520 cP (Δ−0.29, 99% CI [ −0.60, −0.05], *p* = 0.002). This could mean that using information from two modalities instead of one did not, in general, help the observers to discriminate the stimuli. We also compared the multimodal Weber fractions to visual and haptic Weber fractions in isolation. There we saw improvement in discrimination compared to visual 3520 cP (Δ−0.53, 99% CI [−1.00, −0.18], *p* < 10^–5^) and 15 530 cP (Δ−0.83, 99% CI [−2.06, −0.19], *p* = 0.0004).

Perceptual bias due to appearance of opaque and transparent liquids that we observed in task 1c was however significantly reduced (Bonferroni correction for five comparisons α = 0.01, 544 cP: Δ−694.24, 99% CI [−1261.11, −335.42], *p* < 10^–8^; 1100 cP: Δ−625.01, 99% CI [−1028.08, −356.94], *p* < 10^–8^; 1710 cP: Δ−494.88, 99% CI [−747.81, −259.90], *p* < 10^–8^, 4050 cP: Δ−1224.75, 99% CI [−1995.33, −550.91], *p* < 10^–8^) except at the highest viscosity where bias was not significant in task 1c (16 850 cP: Δ−481.66, 99% CI [−2980.42, 1828.98], *p* = 0.30) as shown in figure 4. This indicates that observers were using the haptic information to correct for the bias due to the optical properties of liquids (note that in task 2 the stimuli were not visible while stirring, thus the haptic comparisons cannot depend on stimulus appearance). Among the viscosities with reduced bias, the thin transparent liquid at 544 cP was still perceived as less viscous (Δ124.62, 99% CI [ 3.89, 323.64], *p* = 0.004) while the thicker 4050 cP transparent stimulus was perceived as slightly more viscous (Δ−555.75, 99% CI [−943.91, −150.64], *p* = 0.0004) than opaque stimuli at same viscosity.

In summary, the analysis of Weber fractions in the perception of visual and haptic stimuli showed a slight violation of Weber’s law only for transparent visual stimuli. As the sensitivity to differences in viscosity did not differ significantly between opaque and transparent stimuli, the evidence for this violation is rather weak and would require further investigation. The change of appearance of the liquid in visual perception however led to a significant bias in perceived viscosity, especially at lower viscosities. This bias was significantly reduced when participants were able to stir the liquids, combining visual and haptic information. Discrimination ability for liquids did not improve across the viscosity spectrum compared to unimodal discrimination when participants were able to combine visual and haptic information.

There are many possible explanations for the pattern of behaviour seen when observers judged the viscosity using vision and touch, namely, the reduction in bias, combined with a change in Weber fractions that does not appear to follow the expectation of better stimulus discrimination in a multimodal setting. One possibility is that the observers relied more on the haptic sense and ignored vision. There is however some evidence that participants did not completely discard the visual information: a comparison of the Weber fractions from task 2 (haptic) and task 3a (multimodal with transparent visual stimuli) shows a significant difference at 3520 cP, and a smaller amount of bias persists at the lowest viscosity. A likely explanation is that some interaction between the senses occurred but was dependent on a particular stimulus condition.

## 4. Discussion

While Tiest *et al*. [10] found evidence of violation of Weber’s law across the range of viscosities they tested in the haptic domain, our data suggests that perceptual discrimination tends to scale in proportion to stimulus intensity. While our finding is inconclusive due to deviations in perception of transparent stimuli, it would be interesting if confirmed by future experiments. Tiest *et al*. [10] discuss sources of variation that might affect the Weber fractions and are related to the mode of interaction, for example, the turbulence created when stirring the stimulus in a jar. If Weber fractions are not affected by viscosity, perception has to be able to compensate for these variations, either by explaining them away or by relying on some intrinsic rheological percepts that are yet to be discovered.

Looking at the transparent stimuli in isolation, visual discrimination was comparable or better at low and medium viscosity ranges compared to the highest reference viscosity. This might well be due to the characteristics of transparent stimuli where light travels deeper through the material with little diffusion and refracts at the medium surface, revealing the structure of the environment as flowing, high contrast patterns. As the flow of high viscosity stimuli had lower speed magnitudes, this effect might be due to a decreased discrimination of the speed of these patterns at lower speeds. However, data from literature suggest that visual speed discrimination in natural stimuli is better at lower speeds [28]. A more likely hypothesis, and an interesting one to be tested, is that discrimination is worse at higher viscosity due to more variance in the dynamic shape of the liquid, leading to uncertain information about the overall motion.

The significance and the effect size of the perceptual bias driven by the optical properties of liquids, with transparent liquids on average perceived as less viscous than opaque stimuli (figure 4) suggests that appearance might be an important factor in perception of viscosity. We show that the bias is unlikely due to any prior association with the dispensing of the liquids from a pump (figure 5). Since the addition of the pigment does not affect the physical properties of the fluids, apart from a slight increase in viscosity that we accounted for, the most likely explanation for the bias is the difference in the visual appearance of the liquids. The magnitude of this effect is particularly striking because the influence of visual appearance on viscosity judgements in dynamic stimuli was previously thought to be minimal when reliable motion or shape cues are present [23]. By previous accounts, features describing shape and motion contribute to the perception of viscosity, while appearance mostly affects the recognition and inference of other properties of material such as stickiness or wetness [23]. Mid-level information describing motion and shape was also captured by features in a deep neural network that mimicked the human ability to discriminate viscosity [29]. Our results however suggest that the perceptual shift in viscosity estimates caused by a change in appearance is significant and must be considered in any formal model that accounts for human perception.

The vastly different reflectance properties of opaque and transparent stimuli convey different amounts of information about liquid motion and shape, and this could be an important mediator for the perceptual bias. For example, transparent stimuli could lack reliable motion information and negatively affect both the precision and accuracy of viscosity estimates, thus introducing a bias that is seemingly driven by optical cues, but mainly determined by the measurements of local and global motion. Similarly, opaque stimuli might lack information on shape compared to the rich cues from light aberration in transparent material. While we cannot fully reject this possibility, a few pieces of evidence suggest that the differences in available motion and shape cues are not the only contributing factor to the appearance bias. First, previous work on artificial stimuli [23] found no evidence of difference in available motion and shape information for transparent stimuli (water-like) or opaque (milk-like), as observers could reliably match viscosities in both cases. Second, if the appearance information would provide qualitatively different cues to liquid motion and shape that translate to viscosity, the ability to discriminate stimuli of different viscosity but same appearance would differ between opaque and transparent stimuli. Our measurements of Weber fractions for opaque and transparent stimuli however show no difference in the ability of participants to discriminate between viscosities. This suggests that, when comparing them side by side in the asymmetric opaque-transparent condition, the two stimuli carried a similar amount of information on physical viscosity that could not significantly affect the bias. We leave it to future work to fully rule out or quantify the mediating effect of appearance-based motion estimation.

When observers in our tasks used both visual and haptic information (visuo-haptic study), the discrimination performance did not always improve. This is interesting, as interactions between senses are thought to enhance the precision of discrimination, relative to decisions based on a single sensory modality. Recent research suggests that perceptions of some material qualities are integrated across different sensory systems, for example, when judging gloss [30], softness [31] or roughness [32] using both vision and touch. However, the information might not always be combined across senses, in particular when the perceiver cannot efficiently determine if the signals have the same cause [33]. If the signals come from different sources, they clearly should not be integrated, leading to a complete or partial sensory dominance by one of the cues [34].

Several models of sensory interaction were proposed in the literature, from optimal integration [34,35,36], where independent signals are combined in an information-optimal way, to probability matching, where decisions are based on combining multiple sensory estimates. However, these models base their integration on whether the stimuli are congruent. The performance measured in our work might be therefore explained by the visual stimulus being located on the screen while its physical counterpart was being stirred in a jar, indicating two spatially non-congruent sources of information. The unnaturalness of the visual stimuli could be another reason for the partial haptic capture. The nature of the task might lead to decisions being several steps removed from perceptual cue integration and influenced by many other factors, in contrast to perceptual experiments with more basic stimuli and short exposure times. However, the (spatially) incongruent haptic and visual experience in our tasks is not too far from how liquids are often perceived in real situations. This, together with the persistence of a small but significant appearance bias at the lowest viscosity, as well as a slight improvement in one of the Weber fractions, suggests that the visual cue was taken into account.

A careful experimentation and modelling approach that would include empirical distributions (priors) of appearance and viscosity could further test the mode of integration, using probabilistic models of cue combination. However, recent critiques of testing cue integration in a pooled population dataset should inform any future inquiries in this direction [37,38].

### 4.1. Shortcomings and limitations of experimental studies and analysis

While our method of presenting the stimuli certainly has drawbacks, it is important to note that computer-generated stimuli used in previous studies have their limitations as well. Different algorithms for fluid simulations give visibly different results, where some properties, for example fluid interaction due to surface tension, might not match the real behaviour of materials [39].

We note there are other factors in our tasks that could affect the visual perception of the stimuli beyond the variables of interest. Our videos were not perfectly uniform and included slight variations in the composition and depth of field. The slight variation in the appearance of the plate could, for example, make some of the stimuli more salient and more likely to be selected. There was however no systematic variation across stimuli, and as such we assume that this effect is mostly expressed as noise that does not affect the direction of the measured bias. Displaying only the part of the video, excluding the plate and runoff, confirmed the main result, with transparent stimuli perceived as less thick even when observing only the dispensing from the nozzle (electronic supplementary material, figure S1). Blurriness is another artefact that might affect the perception of stimuli. For example, blurriness might differentially affect stimuli with different spatiotemporal frequencies in the texture. In our videos, the flowing materials are primarily in focus, with pronounced blurriness in the background. As such, we feel that the limited depth of field minimally affects the appearance of the materials.

We also note that in all tasks, the participants were allowed to freely move their upper body during observation, which creates a setup that is closer to a free viewing situation. This introduces a variation in viewing parameters that is much larger that in a setup with controlled fixation. We chose this setup rather deliberately with the intention to make the multimodal task closer to a real-world interaction.

In addition, the particular choice of specific illumination, contrast and background could potentially cause the reported effects while not generalizing to other conditions and stimuli. Further experimentation will be necessary to investigate to what degree our findings generalize, including varying the illumination, stimulus opacity, camera angle, video resolution, and a more varied repertoire of stimulus behaviours.

Measurements might also be affected by prior associations of liquids and the pump dispenser, and possible changes to the flow of the liquids due to the pigment. We have shown through figure 5 and the accompanying discussion that transparent and opaque products on the market are similarly distributed across viscosity, leading to, on average, similar empirical priors. Furthermore, the distribution of viscosities of the consumer products falls almost completely within our stimulus set. At viscosities beyond 2100 cP (at 200 s^−1^ shear rate which is appropriate given the nozzle opening and liquid flow rate), there are very few or no products on the market that use the same pump. Eventual prior expectations would therefore induce similar response biases for the opaque and transparent stimuli. Yet, an association between liquid appearance and common ways of manipulating liquids is still possible and should be taken into account in future work.

Adding pigment might affect the flow in an unforeseen manner, and therefore the perception of stimuli. We verified that all opaque liquids had identical flow properties compared to the transparent liquids, i.e. they show Newtonian behaviour with only a slight increase in viscosity. This small difference in viscosity was considered in the analysis and could not affect the results.

Finally, the way that participants integrated the haptic and visual cues might be due to the different ways stimuli were observed. In the haptic condition, participants actively explored, while in the vision condition, they passively observed fixed-perspective videos. We aimed to mitigate this by using videos that captured multiple phases of liquid behaviour (ejection, free fall, landing and spreading) and allowed sufficient observation time for participants to make an informed decision. While we believe that, given the time of exposure and the richness of the stimulus, participants’ decision would not change drastically, active exploration in vision may still yield different perceptual outcomes, potentially reducing bias. Future studies should further explore this question, balancing exploration across modalities.

### 4.2. Relevance for applied perception

Overall, this work contributes to the understanding of consumer perception when touching and looking at consumer products. Perception of viscosity is an important aspect of product design and must be considered along with stability and performance when designing a product. Designing viscosity for stability and performance can be accomplished through laboratory testing; however, designing viscosity for specific human perception targets requires a knowledge of how humans perceive viscosity in varying modalities, and the interplay of perception in multiple modalities.

For example, human perception of viscosity and other physical properties determine the pleasantness of the consumer experience as intended by the manufacturer. These properties should be such that continued use is desired by the consumer or patient: medicine taken orally or applied topically should elicit positive user responses to ensure compliance and adherence to recommended usage guidelines [40]. A detailed understanding of how humans perceive viscosity and other physical properties is essential to quickly and efficiently design products that are not only effective but also elicit a positive response during use. However, changes in product viscosity due to raw material changes throughout a product’s life cycle are inevitable. If we know how humans perceive viscosity in terms of JNDs for specific modalities of interest, we can reformulate with confidence knowing that even though a reformulated product might exhibit a viscosity that is measurably different compared to the previous version or target, it will not be reliably perceived as different if it lies within the JND of the target, thus reducing the likelihood of alienation and non-compliance.

## Data Availability

Raw data from all experiments is available at [41]. Supplementary material is available online [42].
